# Clinical Outcomes Following Definitive Concurrent Chemoradiotherapy in Patients With International Federation of Gynecology and Obstetrics (FIGO) Stage IB-IVA Carcinoma of the Cervix

**DOI:** 10.7759/cureus.113396

**Published:** 2026-07-26

**Authors:** Sadia Firdous, Fariha Ejaz, Makiya Sharif, Sumbal Hammad, Asia Firdous, Syeda Mehak Mazhar, Ayesha Ahmad, Talha Laique

**Affiliations:** 1 Department of Obstetrics and Gynecology, Medway Maritime Hospital, Gillingham, GBR; 2 Department of Diagnostic Radiology, Islamabad Diagnostic Centre, Lahore, PAK; 3 Department of Obstetrics and Gynecology, Mayo Hospital, Lahore, PAK; 4 Department of Obstetrics and Gynecology, Jinnah Hospital, Lahore, PAK; 5 Department of Pharmacology, Lahore Medical & Dental College, Lahore, PAK; 6 Department of Community Medicine, Azra Naheed Medical College, Lahore, PAK; 7 Department of Radiation Oncology, Institute of Nuclear Medicine and Oncology Lahore (INMOL), Lahore, PAK; 8 Department of Medicine, Mayo Hospital, Lahore, PAK

**Keywords:** cervical carcinoma, concurrent chemoradiotherapy, figo stages, histology patterns, treatment response

## Abstract

Background and aim

Cervical malignancy among women is associated with substantial mortality and morbidity. This study aimed to determine the clinical outcomes of definitive concurrent chemoradiotherapy (CCRT) in patients with International Federation of Gynecology and Obstetrics (FIGO) stage IB-IVA cervical carcinoma.

Methods

This was a prospective, single-arm, interventional (experimental) study. Patients with cervical carcinoma fulfilling the inclusion criteria were planned for CCRT with weekly cisplatin injections (40 mg/m²). Patients with biopsy-proven squamous cell or adenosquamous carcinoma of the cervix, aged 25-65 years, with node-negative (N0) or node-positive (N1) nonmetastatic (M0) disease were included. However, patients with multiple comorbidities, Eastern Cooperative Oncology Group performance status III/IV, previous treatment for cervical cancer, or a history of any other malignancy were excluded. Treatment planning for all patients was performed using a conventional 2D simulator, and patients were treated with 45 Gy external beam radiotherapy (RT) in 25 fractions. A parametrial boost was given after 45 Gy, up to 60 Gy, in patients with parametrial involvement. Data were analyzed using IBM SPSS Statistics for Windows, version 25.0 (released 2017; IBM Corp., Armonk, NY, USA), and the Fisher-Freeman-Halton exact test was applied to compare response rates among patients with different pretreatment FIGO stages. Fisher’s exact test was used to compare response rates after treatment among different histological types. A p-value ≤0.05 was considered significant.

Results

Complete response was observed in 53 (42.7%) patients, while partial response was seen in 45 (36.2%) patients. Stable disease was present in 17 (13.8%) patients, while progressive disease was seen in nine (7.3%) patients. There was a significant difference in response rate across different pretreatment FIGO stages. In our clinical setting, routine human papillomavirus screening and vaccination are not uniformly implemented because of limited resources, inadequate organized screening programs, low public awareness, and financial constraints. This study evaluated the clinical outcomes of definitive CCRT in patients with cervical carcinoma in our population.

Conclusions

Radical CCRT with weekly cisplatin demonstrated favorable short-term treatment outcomes in patients with FIGO stage IB-IVA cervical carcinoma. Most patients achieved a favorable treatment response, supporting acceptable locoregional disease control.

## Introduction

Cervical cancer accounts for 3.3% of all cancers globally (662,301 new cases in 2022) and approximately 2.5% of all female cancers in Pakistan (5,008 new cases; ASR 4.7/100,000 women); about 25% of the global cervical cancer burden occurs in South Asia, with India contributing nearly 19% of worldwide incident cases [1]. Globally, it is the second most common cancer, causing more than 200,000 deaths annually in Asian countries [2]. The inconsistency in mortality due to cervical carcinoma between developing and developed countries is due to poor Pap smear testing [3]. It is primarily caused by persistent infection with high-risk human papillomavirus (HPV), particularly HPV-16 and HPV-18, while smoking, early onset of sexual activity, multiple sexual partners, immunosuppression (e.g., HIV infection), high parity, prolonged oral contraceptive use, and coexisting sexually transmitted infections are recognized contributory risk factors. It has been reported that the HPV vaccine will substantially reduce its morbidity and mortality, as HPV causes 99.7% of invasive cervical cancer cases [4]. This virus is sexually transmitted to humans. Its infections peak in the 18- to 30-year age group [4].

According to the literature, cervical cancers and their precursor lesions contain HPV DNA in almost 99% of cases [5]. Moreover, HPV gene products result in malignant transformation of the epithelium from its normal form, as seen in cervical cancer [6]. HPV-16 contributes to up to 50% of cervical cancer cases and has therefore been a focus of vaccine development [7].

Treatment of cervical cancer depends on the International Federation of Gynecology and Obstetrics (FIGO) stage, disease extent, and patient fitness [8]. Early-stage disease is usually managed with radical surgery or definitive radiotherapy (RT), whereas definitive concurrent chemoradiotherapy (CCRT) followed by brachytherapy is the standard treatment for locally advanced cervical cancer (FIGO IB3-IVA). Patients with recurrent or metastatic disease may require systemic chemotherapy, immunotherapy, targeted therapy, or palliative RT. Among these options, CCRT remains the preferred approach for locally advanced disease because it provides superior survival and locoregional disease control compared with RT alone.

In 2022, Paul et al. evaluated the outcomes of radical RT versus CCRT in patients with locally advanced cervical cancer. The overall five-year response rate was 67% and 53% for the CCRT and RT arms, respectively, and the toxicity profiles were comparable. Hence, the authors concluded that CCRT was better tolerated and more effective than RT alone in the Chinese population [9].

In 2022, Shimels et al. conducted a systematic review and meta-analysis to investigate the outcomes of CCRT in patients with stage IIB-IVA disease. A total of 118 patients with squamous cell carcinoma were enrolled. The five-year overall survival and distant disease-free survival rates for all patients were 64.0% and 78.8%, respectively. The study concluded that CCRT is an effective treatment for cervical cancer [10].

In our clinical setting, routine HPV screening and vaccination are not uniformly implemented because of limited resources, inadequate organized screening programs, low public awareness, and financial constraints. Most patients therefore present with advanced disease. The rationale of our study was to assess the efficacy of CCRT in our patients by evaluating locoregional control of the treatment. At the conclusion of this research, we determined the outcomes of concurrent therapy in our population with cervical carcinoma.

This study provided local evidence on the effectiveness of CCRT with weekly cisplatin in patients with stage IB-IVA cervical carcinoma. The findings demonstrate satisfactory locoregional control, with complete response achieved in a substantial proportion of treated patients, supporting the role of CCRT as an effective treatment strategy for locally advanced cervical cancer in resource-limited settings. The results supported the use of CCRT as a standard treatment approach for locally advanced cervical cancer, offering favorable treatment response and survival outcomes. These findings can assist clinicians in treatment planning, patient counseling, and optimizing management strategies while highlighting the need for improved cervical cancer screening and vaccination programs.

## Materials and methods

Nonmetastatic cervical cancer patients were enrolled through consecutive non-probability sampling in this prospective, single-arm, interventional (experimental) study conducted at the Department of Clinical Oncology, Institute of Nuclear Medicine and Oncology Lahore (INMOL) Hospital, Lahore. After ethical approval of the study, informed consent was obtained.

This study included 124 newly diagnosed nonmetastatic cervical cancer patients aged 25-65 years with histologically confirmed squamous cell or adenosquamous carcinoma of the cervix. Eligible patients had FIGO stage IB3-IVA, node-negative (N0) or node-positive (N1), nonmetastatic (M0) disease and were recruited consecutively from the Radiation Oncology outpatient department after obtaining ethical approval and written informed consent. Patients with multiple significant comorbidities, Eastern Cooperative Oncology Group (ECOG) performance status III/IV, prior treatment for cervical cancer, or a history of another malignancy were excluded [11]. Baseline evaluation included a detailed clinical history, physical and gynecological examination, ECOG performance assessment, and routine laboratory investigations comprising complete blood count, renal and liver function tests, and serum electrolytes. Pretreatment staging was performed using the FIGO staging system, with contrast-enhanced MRI of the pelvis, CT scans of the chest and abdomen, and bone scan where clinically indicated.

All patients received definitive CCRT consisting of weekly intravenous cisplatin (40 mg/m²) administered concurrently with 45 Gy external beam RT (EBRT) in 25 fractions using a conventional 2D treatment planning system. Patients with persistent parametrial involvement received an additional parametrial boost to a total dose of 60 Gy, followed by high-dose-rate (HDR) intracavitary brachytherapy delivering 7 Gy in three fractions according to institutional protocols. Patients were subsequently followed clinically and radiologically to evaluate treatment response using standard clinical and imaging criteria, and treatment outcomes were correlated with baseline demographic and disease characteristics. After a follow-up period of three months, repeat MRI assessment was performed for all patients. The Response Evaluation Criteria in Solid Tumors (RECIST) version 1.1 were used for assessment of response [5]. Patients with FIGO stage IB3-IVA, nonmetastatic (M0), and N0/N1 disease were selected because definitive CCRT is the standard treatment for locally advanced, nonmetastatic cervical cancer. Excluding patients with severe comorbidities, poor performance status, prior treatment, or previous malignancies minimized potential confounding factors and allowed a more accurate assessment of treatment outcomes.

Data were analyzed using IBM SPSS Statistics for Windows, version 25.0 (released 2017; IBM Corp., Armonk, NY, USA). Continuous variables were expressed as mean ± standard deviation, while categorical variables were presented as frequencies and percentages. The Fisher-Freeman-Halton exact test was used to assess the association between pretreatment FIGO stage and treatment response. Fisher’s exact test was used to compare the response rate after treatment among different histology results. A two-tailed p-value ≤0.05 was considered statistically significant.

## Results

The baseline characteristics of the study population are presented in Table 1. The majority of patients with cervical carcinoma had squamous cell carcinoma, whereas the fewest had adenosquamous carcinoma.

**Table 1:** Baseline characteristics of the study population. EBRT, external beam radiotherapy; FIGO, International Federation of Gynecology and Obstetrics; PS, per speculum; PV, per vaginum; RT, radiotherapy

Variable	Category	n (%)
Pretreatment locoregional disease FIGO stage on PV/PS/MRI	1B1	15 (12.0)
	IB2	17 (13.7)
	IIA	18 (14.5)
	IIA1	11 (8.8)
	IIA2	12 (9.6)
	IIB	16 (12.9)
	IIIB	14 (11.2)
	IVA	21 (16.9)
Histology	Squamous	87 (70.1)
	Adenocarcinoma	28 (22.5)
	Adenosquamous	9 (7.2)
RT dose	EBRT + brachytherapy	50 (40.3)
	Parametrial + EBRT + brachytherapy	74 (59.6)

Results showed complete response in 53 (42.7%) patients, while partial response was seen in 45 (36.2%) cases, as shown in Table 2. Stable disease was present in 17 (13.8%) patients, while progressive disease was seen in nine (7.3%) patients.

**Table 2:** Treatment response.

Category	n (%)
Complete response	53 (42.7)
Partial response	45 (36.2)
Stable disease	17 (13.8)
Progressive disease	9 (7.3)

The majority of patients showed a good response to treatment, with a high frequency compared with poor response among the enrolled patients in the current study. The overall good response rate was 104 (83.8%), as shown in Table 3.

**Table 3:** Treatment response according to RECIST. RECIST, Response Evaluation Criteria in Solid Tumors

Category	n (%)
Good response	104 (83.8)
Poor response	20 (16.2)

One of the major strengths of the present study was the evaluation of treatment response across individual pretreatment FIGO stages rather than grouping patients into broad disease categories. This stage-wise analysis enabled a more detailed assessment of the effectiveness of CCRT and identified variations in response according to disease extent. These findings provided clinically relevant evidence for prognostication and treatment planning in patients with cervical carcinoma. The response rate differed significantly across pretreatment FIGO stages according to the Fisher-Freeman-Halton exact test (exact p = 0.021), as shown in Table 4.

**Table 4:** Response rate of patients with different pretreatment FIGO stages. * Statistically significant if p < 0.05 The Fisher-Freeman-Halton exact test, an extension of Fisher’s exact test for contingency tables larger than 2 × 2, was used to assess the association between pretreatment FIGO stage and treatment response. FIGO, International Federation of Gynecology and Obstetrics

FIGO stage	Response, n (%)		Total, n (%)	p-value
	Good	Poor		
1B1	12 (80.0)	3 (20)	15 (100)	0.021*
IB2	14 (82.4)	3 (17.6)	17 (100)	
IIA	15 (83.3)	3 (16.7)	18 (100)	
IIA1	11 (100)	0 (0)	11 (100)	
IIA2	12 (100)	0 (0)	12 (100)	
IIB	13 (81.3)	3 (18.7)	16 (100)	
IIIB	9 (64.3)	5 (35.7)	14 (100)	
IVA	18 (85.7)	3 (14.2)	21 (100)	
Total	104 (83.8)	20 (16.2)	124 (100)	

Another important strength of the present study was the comparison of treatment response across different histological subtypes of cervical carcinoma. By evaluating squamous cell carcinoma, adenocarcinoma, and adenosquamous carcinoma separately, the study assessed whether tumor histology influenced the effectiveness of CCRT. This analysis enhances the clinical applicability of the findings by demonstrating the consistency of treatment outcomes across the major histological subtypes of cervical cancer. Results revealed no significant difference in response rate among the different histology groups, as shown in Table 5.

**Table 5:** Response rate after treatment among different histology results. Fisher’s exact test, an exact statistical test used to assess the association between categorical variables when sample sizes or expected cell counts are small, was used to compare treatment response among different histological subtypes.

Histology	Response, n (%)		Total, n (%)	p-value
	Good	Poor		
Squamous	76 (87.3)	11 (12.6)	87 (100)	0.143
Adenocarcinoma	21 (75.0)	7 (25.0)	28 (100)	
Adenosquamous	7 (77.8)	2 (22.2)	9 (100)	
Total	104 (83.8)	20 (16.1)	124 (100)	

## Discussion

Cervical cancer remains a major public health challenge worldwide, with a substantial disease burden in South Asia, including Pakistan [1]. It is also a leading cause of cancer-related mortality among women in the region [2]. Therefore, patients with cervical carcinoma were treated with radical CCRT to evaluate its efficacy in our population. No deaths were reported in our cohort. Pakistan has a high burden of this disease among females. According to one study, genetic factors, environmental factors, radiation exposure, a low literacy rate, multiple sexual partners, poor menstrual hygiene, and limited awareness of the disease and its consequences all contribute to its high prevalence [12].

The current study enrolled 124 patients, whereas another study enrolled 174 patients to determine the clinical outcomes of patients with advanced cervical cancer receiving CCRT [13]. In contrast, another study enrolled only 87 patients with cervical cancer [9]. Patients received RT daily for five consecutive weeks using a linear accelerator, with 2 Gy delivered per fraction without any planned interruption. Tumor response was assessed three months after treatment completion using CT scans. Cisplatin (40 mg/m²) was administered weekly concurrently with RT. Our findings were consistent with those of a previous study involving patients with head and neck cancer receiving RT [14].

Treatment response was assessed following completion of CCRT, and the distribution of complete response, partial response, stable disease, and progressive disease is presented in Table 2. Our results differed from those of one study, which reported a complete response rate of up to 95.5% among its enrolled patients [15]. This difference may be attributable to genetic differences, radiation dose, treatment technique, radiation schedule, and poor patient compliance. In the current study, disease remained stable in 17 (13.8%) patients, whereas progressive disease was observed in nine (7.3%) patients (Table 2). Similarly, one previous study reported stable disease in 13% of patients, whereas disease progression was observed in only 3% [16].

CCRT with weekly cisplatin remains the standard treatment for patients with locally advanced cervical cancer (FIGO stages IB-IVA). In the present study, the majority of patients demonstrated a favorable treatment response according to RECIST version 1.1 criteria, whereas only a minority exhibited a poor response, as detailed in Table 3. These findings are consistent with published studies reporting objective response rates of 75-90% following definitive cisplatin-based CCRT and support current international guidelines recommending concurrent cisplatin with EBRT followed by brachytherapy as the standard of care for locally advanced cervical carcinoma [17]. CCRT was generally well tolerated; however, common adverse effects included hematologic toxicity, nausea and vomiting, radiation-induced cystitis, diarrhea, fatigue, and mild dermatitis. No treatment-related mortality was reported.

The favorable response observed in this study may be attributed to standardized treatment delivery with weekly cisplatin, EBRT, HDR brachytherapy, and MRI-based response assessment. Nevertheless, a proportion of patients experienced persistent or progressive disease, which may be related to advanced tumor burden, nodal involvement, tumor hypoxia, anemia, or prolonged treatment duration.

Limitations

The main limitations of this study include its single-center design, the absence of a comparison group, the use of conventional 2D RT planning, and a short follow-up period. For logistical reasons, this was a single-center study that lacked PET imaging and genetic testing to evaluate genetic variability among the enrolled patients. Due to logistical constraints, we did not evaluate long-term survival, recurrence patterns, or late treatment-related toxicities. The lack of routine HPV screening and vaccination in our clinical setting, primarily because of limited resources, inadequate organized screening programs, low public awareness, and financial constraints, also represented an important limitation of the present study. However, larger prospective studies with longer follow-up and modern image-guided RT techniques are warranted to evaluate long-term survival and disease control.

## Conclusions

Radical CCRT with weekly cisplatin demonstrated favorable short-term treatment outcomes in patients with FIGO stage IB-IVA cervical carcinoma. A favorable treatment response was observed in the majority of patients, suggesting that CCRT may provide acceptable locoregional disease control in patients with locally advanced cervical cancer. No treatment-related mortality was observed during the study period. These findings support CCRT as an effective treatment modality for locally advanced cervical cancer. However, larger multicenter studies with longer follow-up are warranted to evaluate long-term survival, recurrence patterns, and late treatment-related toxicities.
